# Multiplexed Detection of Cancer Biomarker Using a Dual-Mode Colorimetric-SERS Lateral Flow Immunoassay Based on Elongated Rod Ag Nanoshell (ERNS) SERS Tags

**DOI:** 10.3390/bios16020129

**Published:** 2026-02-21

**Authors:** Sungwoo Park, Yeonghee Jeong, Sohyeon Jang, Cho-Hee Yang, Jun-Sik Chu, Homan Kang, Seung-min Park, Hyejin Chang, Bong-Hyun Jun

**Affiliations:** 1 Department of Bioscience and Biotechnology, Konkuk University, Seoul 05029, Republic of Korea; tjddn939@konkuk.ac.kr (S.P.); jyh3077@konkuk.ac.kr (Y.J.); thgus03030@konkuk.ac.kr (S.J.); vltizk0052@konkuk.ac.kr (C.-H.Y.); cjs9719@naver.com (J.-S.C.); 2 Center for Inflammation Imaging, Department of Radiology, Mass General Brigham and Harvard Medical School, Boston, MA 02114, USA; hkang7@mgh.harvard.edu; 3 School of Chemistry, Chemical Engineering and Biotechnology, Nanyang Technological University, Singapore 637459, Singapore; park.seungmin@ntu.edu.sg; 4 Division of Science Education, Kangwon National University, Chuncheon 24341, Republic of Korea; hjchang@kangwon.ac.kr

**Keywords:** surface-enhanced Raman spectroscopy, Raman labeling compound, SERS tags, lateral flow immunoassay, SiO_2_@Ag, colorimetric, multiplex biomarker detection

## Abstract

Early detection of cancer biomarkers in blood is critical for improving patient outcomes; however, conventional immunoassays often rely on complex instrumentation and are not well suited for point-of-care testing or multiplexed analysis. Herein, we present a dual-mode colorimetric–surface-enhanced Raman scattering (SERS) lateral flow immunoassay (LFIA) platform for multiplexed detection of cancer biomarkers, employing elongated rod-shaped silver nanoshells (ERNSs) as SERS nanotags. The ERNS features a rough Ag shell with internally incorporated Raman labeling compounds (RLCs), enabling plasmonic extinction for visual readout and strong SERS signals for quantitative analysis while preserving the external metal surfaces for efficient antibody conjugation. Leveraging these advantages, a multiplex LFIA capable of simultaneously detecting prostate-specific antigen (PSA) and carbohydrate antigen 19-9 (CA19-9) on a single strip was successfully demonstrated. Visual inspection enabled rapid discrimination of samples at or near clinically relevant cut-off levels, while Raman analysis achieved limits of detection of 8.0 × 10^−3^ ng/mL for PSA and 5.4 × 10^−2^ U/mL for CA19-9, corresponding to approximately 500-fold and 685-fold lower concentrations than their respective clinical thresholds. This ERNS-based colorimetric–SERS LFIA integrates rapid screening and highly sensitive quantification within a single platform and offers a versatile nanoprobe design strategy for multiplex biomarker detection and liquid biopsy-based diagnostic applications, with potential relevance to point-of-care settings.

## 1. Introduction

Early cancer diagnosis is a critical determinant of patient survival and therapeutic outcomes [1,2,3,4]. While liquid biopsy approaches based on circulating protein biomarkers offer minimally invasive alternatives to tissue biopsy [5,6,7,8], there is an increasing clinical demand for analytical platforms capable of the simultaneous detection of multiple, disease-specific biomarkers, particularly for enabling parallel screening across different cancer types rather than reliance on a single indicator [9,10,11,12,13,14]. However, achieving multiplexed and quantitative biomarker detection in point-of-care testing (POCT) settings remains challenging, as conventional immunoassays such as ELISA and chemiluminescent immunoassays require complex instrumentation and trained personnel [15,16,17,18,19].

In this context, lateral flow immunoassays (LFIAs) have emerged as attractive alternatives owing to their simplicity, rapid response, and low cost [20,21,22,23,24,25,26]. However, conventional colorimetric LFIAs relying on Au nanoparticle reporters suffer from limited sensitivity and poor quantitative capability, particularly at low analyte concentrations [27,28,29,30,31]. Accordingly, plasmonic nanostructures have been explored to enhance visual sensitivity in colorimetric LFIAs [32,33,34,35], while surface-enhanced Raman scattering (SERS)-based approaches employing Raman-tagged nanoparticles have enabled highly sensitive quantitative analysis [36,37,38,39,40], suggesting the potential benefit of dual-mode LFIA platforms that combine rapid visual screening with reliable quantitative readout [41,42,43,44,45,46].

Recently, our group reported silica-templated elongated rod-shaped Ag nanoshells (ERNS) featuring a rough Ag shell and elongate morphology, which exhibited strong and reproducible SERS activity through a simple synthesis route [47]. To date, these ERNS have primarily been utilized for label-free SERS detection via the exposed Ag surface, while their potential as SERS-tagged nanoprobes for immunoassay applications has not been explored. Notably, embedding Raman labeling compounds (RLCs) within the Ag nanoshell provides a clear advantage for bioassays: it yields strong SERS signals while leaving the outer metal surface available for efficient antibody conjugation, which is particularly valuable in multiplex LFIA formats. Here, RLCs refer to Raman-active reporter molecules comprising a metal-anchoring group, typically a thiol moiety for Ag nanostructures, and a Raman-active aromatic framework that provides distinct and identifiable Raman signatures.

**Figure 1:**
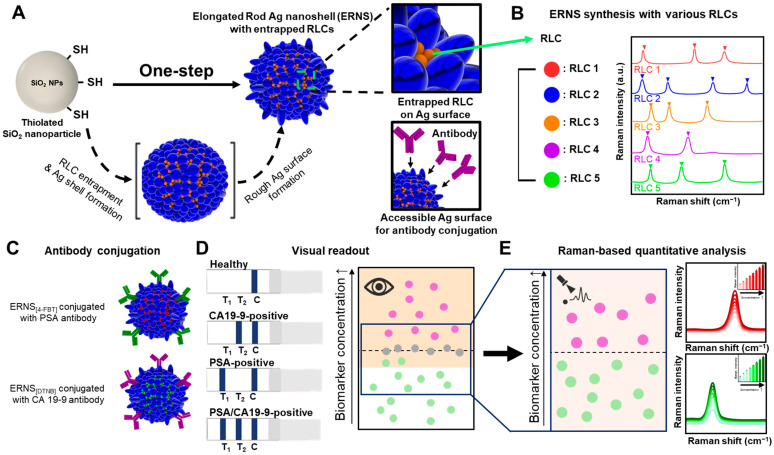
(**A**) Schematic illustration of the one-step synthesis of ERNS on a silica template, where Raman labeling compounds (RLCs) are introduced during Ag shell growth. (**B**) Schematic illustration showing the synthesis of ERNS with different RLCs, indicating the versatility of ERNS as nanoprobes for multiplex detection. (**C**) Schematic illustration of antibody conjugation onto ERNS labeled with different RLCs, enabling specific recognition of PSA and CA19-9. (**D**) Schematic illustration of the application of ERNS-based probes to a SERS-LFIA platform, where colorimetric signals at the test lines allow visual pre-screening of target biomarkers. (**E**) Schematic illustration of Raman analysis performed at the test lines for quantitative detection, enabling accurate classification of samples and detection at ultralow concentrations.

In this study, we report a colorimetric–SERS dual-mode LFIA platform based on ERNS with internally incorporated Raman labeling compounds for the simultaneous detection of prostate-specific antigen (PSA) and carbohydrate antigen 19-9 (CA19-9). Figure 1A schematically illustrates the one-step synthesis of elongated rod-shaped Ag nanoshells (ERNS) on a silica template, in which Raman labeling compounds (RLCs) are incorporated during Ag shell growth. The RLCs are entrapped within the Ag nanoshell, while the outer Ag surface remains accessible for antibody conjugation, enabling stable internal Raman reporting without compromising SERS activity. ERNS possess structural and spectral features that make them well suited as multiplexed SERS reporters, as different RLCs can be incorporated to generate spectrally distinguishable encoding tags without altering particle morphology (Figure 1B). ERNS encoded with distinct Raman labeling compounds were functionalized with biomarker-specific antibodies and integrated into a dual-mode lateral flow immunoassay, enabling multiplexed colorimetric screening and SERS-based identification (Figure 1C). The proposed system enables rapid visual discrimination of samples near clinically relevant cut-off levels (Figure 1D), followed by reliable Raman-based quantitative analysis at low concentration levels (Figure 1E). By integrating complementary visual and SERS readouts within a single LFIA strip, this work demonstrates the practical utility of ERNS-based SERS tags for multiplex liquid biopsy diagnostics in point-of-care settings.

## 2. Materials and Methods

### 2.1. Materials

Ethanol (98%), tetraethyl orthosilicate (TEOS, 99.99%), 3-mercaptopropyl trimethoxysilane (MPTS, 95%), polyvinylpyrrolidone (PVP) (average molecular weight ~40,000), silver nitrate (AgNO_3_, 99.99%), ethylene glycol (EG, ≥99%), hexadecylamine (≥94.0%), 4-fluorothiophenol (4-FBT, 98%), 4-aminothiophenol (ATP, 97%), 4-chlorothiophenol (4-CBT, 97%), 4-mercaptobenzoic acid (4-MBA, 99%), 5,5′-dithiobis(2-nitrobenzoic acid (DTNB, ≥98%), 11-mercaptoundecanoic acid (11-MUA), 1-ethyl-3-(3-dimethylaminopropyl)carbodiimide hydrochloride (EDC), sulfo-N-hydroxysulfosuccinimide (sulfo-NHS), Tween 20, 4-morpholineethanesulfonic acid (MES), ethanolamine, bovine serum albumin (BSA), 96-well plate were purchased from Sigma-Aldrich (St. Louis, MO, USA). Ammonium hydroxide (NH_4_OH, 25–28%) was purchased from Daejung (Siheung, Republic of Korea).

Phosphate-buffered saline (PBS, pH 7.4) and 0.5% (*v*/*v*) Tween 20 in phosphate-buffered saline (PBST, pH 7.4) were purchased from DyneBio (Seongnam, Republic of Korea).

Prostate-specific antigen (PSA) was purchased from Fitzgerald Industries (Acton, MA, USA). Anti-PSA antibodies (Ab 14801 and Ab14803), CA19-9 antigen (J66100) and CA19-9 monoclonal antibodies (A46300 and A46400) were purchased from BiosPacific Inc. (Emeryville, CA, USA). Goat anti-mouse IgG antibody was purchased from Bore Da Biotech Co., Ltd. (Seongnam, Republic of Korea). Nitrocellulose (NC) membrane, backing card, and absorbent pad were purchased from Bore da Biotech Co., Ltd. (Seongnam, Republic of Korea).

### 2.2. Instruments

Centrifuge (LaboGene 1248R, LaboGene Co. Ltd., Daejeon, Republic of Korea). Electron microscopic images were obtained using transmission electron microscopy (TEM; JEM-1010, JEOL Ltd., Tokyo, Japan). UV-Visible spectra were measured using a UV-Vis spectroscopy (Ratio Beam Spectrophotometer U-5100, Hitachi, Ltd., Tokyo, Japan). The SERS performance of nanoprobes was characterized using a Raman microscope (DXR3xi, Thermo Fisher Scientific, Waltham, MA, USA); spectra were recorded in the range of 200–2800 cm^−1^ under a 780 nm laser at 10 mW power, with eight exposures of 2 s each at 10× magnification (total exposure time: 16 s). Raman spectra of the lateral flow immunoassay (LFIA) strips for PSA and CA19-9 detection were measured using a Raman spectrometer (i-Raman Prime 785, B&W TEK, Newark, DE, USA) with a 785 nm laser at 2 mW power. Each Raman system was used exclusively according to its experimental purpose. The DXR3xi Raman microscope was employed only for evaluating the intrinsic SERS performance of the nanoprobes in colloidal dispersion under controlled laboratory conditions, whereas the i-Raman Prime system was used solely for Raman measurements of LFIA strips to reflect point-of-care detection scenarios. Quantitative analyses and calibration curves were established independently within each Raman platform, and no direct signal calibration or quantitative comparison was performed between different Raman instruments. The colorimetric intensity of the commercial LFIA strip was quantified using ImageJ software (version 1.52a).

### 2.3. Synthesis and Surface Thiolation of Silica Nanoparticles

Silica nanoparticles (SiO_2_ NPs) were prepared via a modified Stöber process. Briefly, ethanol (40 mL) and NH_4_OH (4.5 mL) were introduced into a round-bottom flask and mixed at 500 rpm for 5 min in a water bath set to 60 °C. Tetraethyl orthosilicate (TEOS, 1.6 mL) was then added to the reaction mixture, followed by continuous stirring at 700 rpm for 1 h while maintaining the same temperature. Subsequently, the flask was transferred to a water bath at room temperature, and the reaction was allowed to proceed for an additional 3 h under stirring at 700 rpm. After completion of the reaction, monodisperse SiO_2_ NPs were obtained and dispersed in ethanol.

For the preparation of ERNS precursors, surface hydroxyl groups on the SiO_2_ NPs were converted to thiol functionalities through silanization with 3-mercaptopropyltrimethoxysilane (MPTS). In a typical procedure, 12.5 mg of the as-prepared SiO_2_ NPs dispersed in ethanol were mixed with liquid MPTS (12.5 μL, 95%) and NH_4_OH (2.5 μL), followed by incubation at room temperature with continuous agitation at 700 rpm using a microtube mixer overnight. The resulting thiol-functionalized SiO_2_ NPs were purified by centrifugation at 12,000 rcf and 4 °C, with five successive washing steps using ethanol. The final product was redispersed in ethanol and stored for subsequent experiments.

### 2.4. Synthesis of a One-Step ERNS (O-ERNS)

Thiol-functionalized SiO_2_ NPs (2 mg) were dispersed in 25 mL of ethylene glycol (EG) containing poly(vinylpyrrolidone) (PVP, 5 mg). Subsequently, 25 mL of an AgNO_3_ solution prepared in EG was introduced to the suspension, yielding a final AgNO_3_ concentration of 3.5 mM. To trigger the formation of O-ERNS, a hexadecylamine solution (1 mL) was immediately injected into the reaction system to achieve a final concentration of 5 mM. The mixture was stirred at 700 rpm for 15 min at room temperature, after which 100 μL of a 4-fluorobenzenethiol (4-FBT) solution (1 × 10 ^−6^ M) was rapidly added. The reaction was allowed to proceed for an additional 1 h under continuous stirring at 700 rpm at room temperature. Upon completion, the resulting nanoparticles were collected and purified by centrifugation at 3000 rcf and 4 °C using a centrifuge (LaboGene 1248R, LaboGene Co., Ltd., Daejeon, Republic of Korea), followed by five successive washing steps with ethanol to remove residual reactants.

### 2.5. Synthesis of a Multi-Step ERNS (M-ERNS)

Thiol-functionalized SiO_2_ NPs (2 mg) were introduced into 25 mL of ethylene glycol (EG) containing poly(vinylpyrrolidone) (PVP, 5 mg), followed by the addition of 25 mL of an AgNO_3_ solution to achieve a final AgNO_3_ concentration of 3.5 mM. For the synthesis of M-ERNS, hexadecylamine (1 mL) was rapidly injected into the reaction mixture, resulting in a final concentration of 5 mM. The suspension was then stirred at room temperature for 1 h to allow the formation of the Ag shell. After completion of the reaction, the nanoparticles were purified by centrifugation at 3000 rcf and 4 °C with five successive ethanol washing steps to remove excess reagents.

For post-synthesis functionalization, 0.5 mL of the M-ERNS suspension (SiO_2_ concentration: 0.4 mg/mL) was mixed with 0.5 mL of a 4-fluorobenzenethiol (4-FBT) solution (0.2 mM) and stirred at room temperature for an additional 1 h. The resulting nanoparticles were subsequently washed three to five times with ethanol by centrifugation to ensure complete removal of unbound molecules.

### 2.6. Characterization of Silica NPs Encapsulated with Silver Nanoshells

The size and morphology of the synthesized nanoparticles were examined using transmission electron microscopy (TEM; JEM-1010, JEOL Ltd., Tokyo, Japan). For TEM observation, 20 μL of a nanoparticle suspension with a concentration of 1 mg/mL, dispersed in the colloidal phase, was drop-cast onto a carbon-coated copper grid (Ted Pella Inc., Redding, CA, USA) and allowed to dry at room temperature for 5–10 min prior to imaging.

Quantitative analysis of particle geometry was performed by measuring the circularity and perimeter of 100 randomly selected nanoparticles from the TEM images using ImageJ software (National Institutes of Health, Bethesda, MD, USA). Circularity was calculated according to Equation (1):

(1) $$Circularity\;=\;\frac{4\pi A}{P^{2}}\;\;\;\;\;$$

where *A* denotes the cross-sectional area of an individual nanoparticle, and *P* represents its perimeter. UV–visible extinction spectra were collected using a ratio beam spectrophotometer (U-5100, Hitachi, Ltd., Tokyo, Japan) equipped with a polystyrene cuvette (12.5 × 12.5 × 45 mm, optical path length of 10 mm; Ratiolab GmbH, Dreieich, Germany). Prior to measurement, all samples were diluted 150-fold to a final concentration of 1 mg mL^−1^, and spectra were recorded over a wavelength range of 290–1100 nm.

### 2.7. Antibody Conjugation onto Synthesized ERNS

The surface of the synthesized ERNS was modified with 11-mercaptoundecanoic acid (11-MUA). Briefly, 0.1 mg of ERNS nanoparticles was mixed with 10 μL of 2 mM 11-MUA solution and reacted at room temperature for 1 h. The MUA-modified ERNS were then washed twice with ethanol and twice with deionized water by centrifugation at 8500 rpm for 10 min at 4 °C.

Next, 100 μL of deionized water containing 2 mM 1-ethyl-3-(3-dimethylaminopropyl) carbodiimide (EDC) and 100 μL of deionized water containing 2 mM sulfo-N-hydroxysulfosuccinimide (sulfo-NHS), along with 500 mM MES, were sequentially added to the ERNS dispersion and reacted at room temperature for 30 min to activate the carboxyl groups on the 11-MUA-modified surface. Following activation, the mixture was centrifuged at 15,000 rpm for 10 min at 4 °C, and the supernatant was removed. The resulting pellet was dispersed in 1 mL of 50 mM 2-(N-morpholino)ethanesulfonic acid (MES) buffer.

Then, 10 μL of an antibody solution (1.0 mg/mL) was added to the ERNS suspension, gently mixed, and incubated at room temperature for 2 h. Specifically, ERNS labeled with 4-FBT were conjugated with anti-PSA antibody (Ab14803), while the ERNS labeled with DTNB were conjugated with anti-CA19-9 antibody (Ab46400). After conjugation, the antibody-bound ERNS were washed with 1 mL of 50 mM MES buffer by centrifugation at 17,000 rpm for 10 min at 4 °C. Subsequently, 3.2 μL of ethanolamine was added to the suspension and incubated at room temperature for 30 min to quench unreacted sulfo-NHS esters and minimize nonspecific interactions. Finally, the particles were washed twice each with 0.5% PBST and 0.5% BSA/PBS by centrifugation at 17,000 rpm for 10 min at 4 °C, and then resuspended in 1 mL of 0.5% BSA/PBS. The resulting pellet was stored at 4 °C until further use.

### 2.8. Preparation of Test Strips for the ERNS-Based LFIA

For the preparation of the test strip, the NC membranes were assembled onto backing cards, and goat anti-mouse IgG antibody solution (1 mg/mL) was dispensed onto the control line. For PSA detection, anti-PSA antibody (14801) solution (1 mg/mL in PBS) was dispensed onto the test line, and for CA19-9 detection, CA19-9 monoclonal antibody (A46300) solution (1 mg/mL in PBS) was dispensed onto the test line.

For the preparation of strips for multiplex detection of PSA and CA19-9, anti-PSA antibody (14801) solution was dispensed onto test line 1, and CA19-9 monoclonal antibody (A46300) solution was dispensed onto test line 2. The spacing between each test line and the control line was set at 5 mm.

Both the test and control lines were dispensed at a flow rate of 0.1 mL/min. The antibody-dispensed strips were then dried in a desiccator for 2 h. After drying, an absorbent pad was attached to the backing card, and the strips were cut into 4 mm widths to prepare the final test strips.

### 2.9. Validation of Individual PSA and CA19-9 Detection via SERS-Based LFIA

PSA and CA19-9 antigen solutions were serially diluted in 0.5% PBST to various concentrations. For single detection, 1 μL of PSA Ab–conjugated 4-FBT ERNS probe, 25 μL of antigen solution, and 34 μL of 0.5% PBST (total 60 μL) were sequentially added to the well of a 96-well plate. SERS-LFIA strips with 1 mg/mL of anti-PSA Ab immobilized on the test line were vertically inserted into the wells to initiate the assay. The same procedure was applied for CA19-9 detection using the CA19-9 Ab–conjugated DTNB ERNS probe and CA19-9 antigen solutions.

### 2.10. Application of O-ERNS-Based LFIA for Multiplex Biomarker Detection

For the assay, 25 μL each of PSA and CA19-9 antigen solutions, 1 μL each of the corresponding Ab–conjugated ERNS probes (0.4 mg/mL), and 8 μL of 0.5% PBST were mixed in a 96-well plate to a final volume of 60 μL.

The reaction mixtures were left undisturbed for 20 min to allow sufficient binding between the antigen and antibody, and the prepared dual-mode LFIA strips were vertically inserted. The solution migrated along the strip via capillary action, initiating the immunoassay reaction.

### 2.11. Calculation of Analytical Limit of Detection

Calibration curves were fitted using a four-parameter logistic (4PL) regression model with a variable slope, described by the following Equation (2):

(2) $$Y=\;Bottom+\frac{X^{Hillslope}(Top-Bottom)}{X^{Hillslope}+{EC50}^{Hillslope}}\;\;\;\;\;$$

where *X* is the analyte concentration, and *Y* is the measured response. *Top* and *Bottom* represent the upper and lower asymptotic response plateaus, respectively; *EC*50 is the concentration producing 50% of the maximal response, and *Hillslope* is a dimensionless slope factor.

The limit of detection (LOD) was defined as the analyte concentration corresponding to a response equal to the mean signal of blank samples plus 3.3 times the standard deviation (mean_blank_ + 3.3σ). The corresponding concentration was obtained by inverse interpolation from the fitted 4PL calibration curve.

## 3. Results and Discussion

### 3.1. Structural Evolution and Optical Properties of ERNS During Synthesis

**Figure 2:**
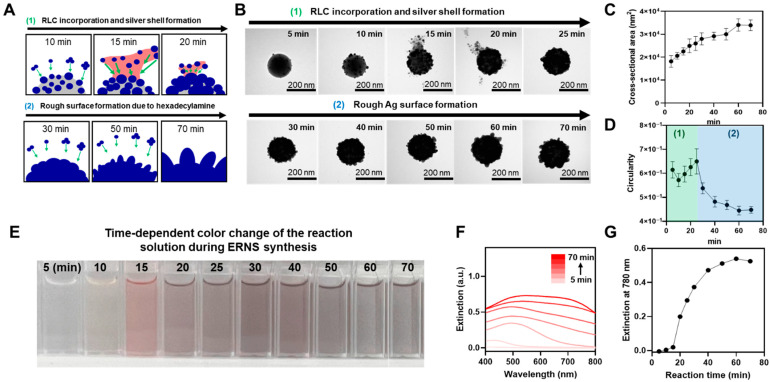
(**A**) Schematic illustration of the proposed ERNS growth process. (**B**) Time-dependent TEM images of ERNS collected at the indicated reaction times during synthesis. (**C**) Cross-sectional area of ERNS as a function of reaction time. (**D**) Circularity of ERNS as a function of reaction time. The green-shaded region (**1**) indicates the reaction period associated with RLC incorporation and Ag shell formation, while the blue-shaded region (**2**) corresponds to the subsequent stage dominated by rough surface development attributed to hexadecylamine-mediated structural evolution. (**E**) Photographs of the reaction solution showing the time-dependent color change during ERNS synthesis. (**F**) UV–vis extinction spectra of the reaction solution recorded at different reaction times. (**G**) Extinction intensity at 780 nm plotted as a function of reaction time.

ERNS were synthesized using a silica template, in which a Ag shell grows along the surface of the template, and their structural formation and growth behavior were systematically investigated. During synthesis, Ag ions are gradually reduced and deposited under hexadecylamine-mediated conditions, leading to the formation of a rough and structurally irregular Ag shell. In this process, thiolated RLCs are introduced during shell growth and become embedded within the developing Ag shell, serving as a key structural feature for SERS applications. While such architectures can provide plasmonic properties favorable for SERS [46], how the Ag shell evolves over synthesis time and how the incorporation behavior of RLCs is coupled with shell growth remains incompletely understood. Accordingly, we monitored the time-dependent morphological evolution of ERNS using time-resolved TEM analysis and analyzed the structural evolution of the Ag shell and the incorporation behavior of RLCs in conjunction with the accompanying changes in optical properties. Figure 2A schematically illustrates the proposed synthesis process of ERNS. Hexadecylamine acts as a reducing agent that moderates the reduction rate of Ag ions, under which Ag undergoes gradual nucleation and growth on the surface of the silica template. Such a relatively slow reduction environment promotes localized and non-uniform Ag shell growth rather than uniform deposition, which is known to contribute to the formation of a rough surface morphology [48].

When Raman labeling compounds (RLCs) are introduced at approximately 15 min, which was identified as the optimal addition time in a prior optimization study [47], strong Ag–S interactions are formed between the growing Ag nanoparticles and the thiol groups of the RLCs, and this process is inferred to give rise to the transient appearance of lamella-like intermediate structures observed in TEM images. These lamella-like intermediates are reasonably attributed to transient Ag–thiolate (Ag–RLC) complexes formed through strong Ag–S coordination, which are gradually reduced and incorporated into the growing Ag shell, thereby facilitating RLC entrapment [49]. This Ag–S–mediated binding process appears to proceed within a relatively short time scale, after which Ag species that are not yet fully reduced continue to grow around these intermediate structures. Meanwhile, the presence of hexadecylamine contributes to the gradual development of nanoscale surface roughness on the Ag shell, and upon completion of the reaction, ERNS with a fully roughened Ag surface are observed.

To experimentally validate the proposed growth mechanism, the time-dependent structural evolution of ERNS during synthesis was monitored by transmission electron microscopy (TEM) (Figure 2B). The growth process of ERNS can be broadly divided into two stages. The first stage corresponds to the initial formation of the Ag shell accompanied by internal incorporation of RLCs, while the second stage involves the development of surface roughness. Immediately after the initiation of the reaction, Ag nucleation and growth on the silica surface were observed. Shortly after the RLC introduction, transient lamella structures appeared due to interactions between Ag and the RLCs. However, these lamella structures were no longer observed after 25 min, indicating that the RLC–Ag binding process is completed within approximately 10 min. In the later stage of the reaction, beginning at around 30 min, the surface roughness of the Ag shell became increasingly pronounced, and nanoscale protrusions and surface irregularities continued to develop over time. At the end of the reaction, ERNS with a fully developed nanoscale rough surface were clearly observed.

Quantitative analysis of the cross-sectional area of nanoparticles extracted from the time-resolved TEM images revealed a continuous increase from the initial stage to the end of the reaction (Figure 2C), confirming that Ag growth proceeds along the shell structure even after RLC incorporation. In addition, circularity analysis of ERNS at corresponding time points showed a characteristic evolution (Figure 2D). Circularity initially decreased due to Ag nanoparticle growth on the spherical silica surface, followed by an increase after RLC introduction as the silica surface became fully covered by Ag nanoparticles. In the later stage, circularity decreased sharply as nanoscale surface roughness rapidly developed on the Ag shell under the influence of hexadecylamine. These results quantitatively demonstrate the time-dependent formation of surface roughness on ERNS. Next, the time-dependent color change in the reaction solution during ERNS synthesis was monitored (Figure 2E). The initially transparent solution gradually changed to yellow and red as Ag nucleation and shell growth proceeded on the silica surface, and eventually appeared nearly black at the end of the reaction. This gradual color evolution visually reflects the continuous evolution of plasmonic properties accompanying Ag shell growth. Consistent with these observations, UV–vis extinction spectra collected at different reaction times exhibited a progressive red-shift and an increase in extinction intensity as the Ag shell formed and thickened (Figure 2F). In particular, quantitative analysis of the extinction intensity at 780 nm showed a continuous increase with reaction time (Figure 2G). This increase in plasmonic extinction indicates enhanced light–nanoparticle interactions and provides favorable optical characteristics for improving visual colorimetric contrast under identical conditions. These results demonstrate that ERNS simultaneously acquire optical properties suitable for colorimetric readout in addition to strong SERS activity during the synthesis process.

### 3.2. Structural and Optical Comparison of ERNS with Different RLC Incorporation Strategies and Versatility Toward Multiple RLCs

**Figure 3:**
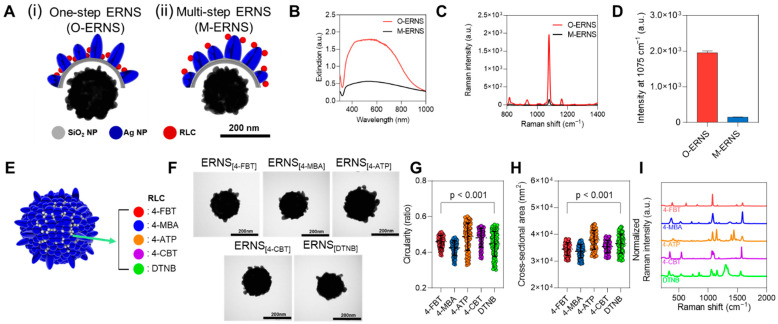
(**A**) Schematic illustrations and TEM images of ERNS synthesized via (i) one-step ERNS (O-ERNS) with internal RLC incorporation and (ii) multi-step ERNS (M-ERNS) with surface-located RLC. (**B**) UV–vis extinction spectra of O-ERNS and M-ERNS measured in the wavelength range of 300–1000 nm. (**C**) Raman spectra of O-ERNS and M-ERNS after incorporation of 4-FBT at a concentration of 10^−2^ M. (**D**) Comparison of Raman intensities at 1074 cm^−1^ corresponding to the characteristic peak of 4-FBT for O-ERNS and M-ERNS. (**E**) Schematic illustration of ERNS synthesis using five different RLCs (4-FBT, 4-MBA, 4-ATP, 4-CBT, and DTNB). (**F**) TEM images of ERNS synthesized with different RLCs. (**G**) Circularity distributions of ERNS synthesized with different RLCs. (**H**) Cross-sectional area distributions of ERNS synthesized with different RLCs. (**I**) Raman spectra of ERNS synthesized with different RLCs, showing the characteristic signatures of each RLC.

Next, the structural and optical differences in ERNS depending on the RLC incorporation strategy, as well as the feasibility of synthesizing ERNS with various RLCs, were systematically compared and analyzed. Specifically, (i) one-step ERNS (O-ERNS), designed to incorporate RLCs into the internal structure by introducing RLCs during Ag shell growth, was compared with (ii) multi-step ERNS (M-ERNS), in which RLCs were introduced in an additional step after completion of Ag shell formation, resulting in RLC localization at the outermost surface (Figure 3A). Both structures exhibited rough Ag shells formed on silica templates; however, distinct structural concepts—internal encapsulation versus surface localization of RLCs—were realized depending on the timing of RLC incorporation. As previously reported, the distinct spatial localization of RLCs in O-ERNS and M-ERNS was experimentally examined by probing the accessibility of the outermost Ag surface using surface accessibility probing model experiments [47]. This established structural distinction provides the basis for the comparative optical analyses presented below. To further support the distinct RLC localization and its consequence on surface accessibility, additional competitive adsorption experiments were conducted using O-ERNS pre-labeled with 4-FBT. First, upon exposure to thiram (1.0 × 10^−3^ to 1.0 × 10^−7^ M), the Raman intensity of 4-FBT remained unchanged compared to the blank control, indicating that the pre-incorporated 4-FBT was not readily displaced by externally introduced thiol molecules (Figure S1A,B). Next, under the same conditions, only the characteristic Raman peaks of thiram increased proportionally with increasing concentration, confirming that the outermost Ag surface of O-ERNS remained accessible for additional thiol binding while preserving the embedded 4-FBT signal (Figure S1C).

To compare the optical properties of these two nanostructures, UV–vis extinction spectra were analyzed. O-ERNS exhibited higher extinction intensities than M-ERNS across the wavelength range of 300–1000 nm (Figure 3B). Subsequently, Raman spectra were compared after incorporation of an identical amount of 4-fluorobenzenethiol (4-FBT). O-ERNS displayed substantially stronger Raman signals than M-ERNS over the entire spectral range (Figure 3C). In particular, quantitative comparison of the Raman intensity at the characteristic 4-FBT peak at 1074 cm^−1^ revealed values of 1954.06 for O-ERNS and 149.24 for M-ERNS, corresponding to a signal enhancement of more than 13-fold (Figure 3D). To further validate the enhanced SERS performance of O-ERNS, a direct comparative experiment was conducted using a bare Ag shell synthesized under identical conditions without RLC incorporation.

When the Raman intensity at the characteristic 4-FBT peak (1075 cm^−1^) was compared, O-ERNS exhibited a 365.93-fold higher signal relative to the bare Ag shell, whereas M-ERNS showed a 27.95-fold increase.

Accordingly, the relative SERS enhancement of O-ERNS over M-ERNS was approximately 13.1-fold (365.93/27.95), which is consistent with the quantitative comparison shown in Figure 3D and Figure S2A,B.

In addition, the feasibility of synthesizing ERNS with various RLCs was evaluated to assess their potential as nanoprobes for multiplex detection (Figure 3E–I). ERNS were synthesized using five different RLCs—4-FBT, 4-mercaptobenzoic acid (4-MBA), 4-aminothiophenol (4-ATP), 4-chlorobenzenethiol (4-CBT), and 5,5′-dithiobis(2-nitrobenzoic acid) (DTNB)—as schematically illustrated in Figure 3E. TEM analysis confirmed that continuous Ag shells were fully formed on the silica templates under all RLC conditions, and that the rough surface morphology was preserved regardless of the RLC employed (Figure 3F). Quantitative analysis of nanoparticle circularity (Figure 3G) and cross-sectional area (Figure 3H) further showed that ERNS synthesized with different RLCs exhibited highly similar distributions and average values, indicating minimal structural variation arising from the choice of RLC. Finally, Raman analysis revealed distinct and characteristic Raman spectra corresponding to each incorporated RLC, confirming their successful incorporation into the ERNS structure (Figure 3I). Collectively, these results demonstrate that ERNS can be synthesized with a variety of RLCs while maintaining structural uniformity and optical specificity, thereby supporting their versatility as SERS-based nanoprobes for multiplex biomarker detection.

### 3.3. Effect of RLC Location on Antibody Binding Behavior and SERS-LFIA Performance of ERNS

**Figure 4:**
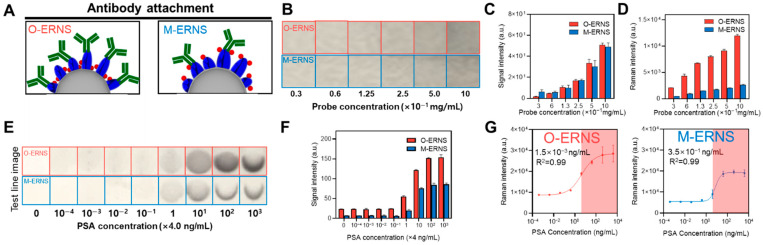
(**A**) Schematic illustration comparing O-ERNS and M-ERNS, highlighting the difference in available Ag surface area for antibody conjugation. (**B**) Colorimetric images of O-ERNS and M-ERNS drop-cast onto nitrocellulose (NC) membranes at concentrations ranging from 10 × 10^−1^ to 0.3 × 10^−1^ mg/mL. (**C**) Quantified colorimetric signal intensities extracted from the images in (**B**). (**D**) Raman intensities measured from the corresponding regions on the NC membrane shown in (**B**). (**E**) Colorimetric images of test lines obtained after applying O-ERNS- and M-ERNS-based SERS-LFIA to PSA samples with concentrations ranging from 0 to 4 × 10^3^ ng/mL. (**F**) Quantified colorimetric intensities of the test lines shown in (**E**). (**G**) Raman intensities measured at 613 cm^−1^ from the test lines at different PSA concentrations and the corresponding limits of detection (LOD).

Next, the effect of RLC localization on the antibody binding behavior of ERNS and their performance in SERS-LFIA was evaluated. Based on the structural differences between O-ERNS and M-ERNS, it was conceptually proposed that O-ERNS possess a relatively larger accessible Ag surface area due to the absence of RLCs at the outermost surface, thereby allowing a higher density of antibody conjugation compared to M-ERNS (Figure 4A).

To investigate how this structural difference influences colorimetric and SERS signals, O-ERNS and M-ERNS were drop-cast onto nitrocellulose (NC) membranes at concentrations ranging from 10 × 10^−1^ to 0.3 × 10^−1^ mg/mL, and the resulting colorimetric images were examined (Figure 4B). Quantitative analysis of the colorimetric signals extracted from these images revealed that O-ERNS and M-ERNS exhibited nearly identical colorimetric intensities across all concentration ranges (Figure 4C), indicating that the localization of RLCs has a negligible effect on the intrinsic colorimetric properties of the nanoparticles. In contrast, Raman analysis performed on the same regions showed that O-ERNS generated substantially stronger Raman signals than M-ERNS at all nanoparticle concentrations (Figure 4D). This result suggests that RLCs positioned within the metal shell are more effectively coupled to plasmonic hot spots than those located at the outer surface, leading to pronounced SERS signal enhancement.

Subsequently, O-ERNS and M-ERNS were applied to an actual SERS-LFIA system, and colorimetric images of the test lines capturing the nanoprobes were compared after running samples with PSA concentrations ranging from 4 × 10^3^ ng/mL to 0 (Figure 4E). Notably, within the clinically relevant concentration range above the PSA cut-off value (4 ng/mL to 4 × 10^3^ ng/mL), O-ERNS produced test lines that were both darker and broader than those obtained with M-ERNS. This behavior is attributed to the higher antibody loading capacity of O-ERNS arising from their larger accessible surface area, which enables a greater number of probes to be captured at the test line at the same PSA concentration. Consistent with these observations, quantitative analysis of the test line colorimetric intensities showed that O-ERNS exhibited stronger signals than M-ERNS across the entire PSA concentration range (Figure 4F).

Finally, Raman analysis was performed on the test line regions, and the corresponding Raman intensities were used to evaluate the analytical performance of the SERS-LFIA system (Figure 4G). The O-ERNS-based SERS-LFIA achieved a limit of detection (LOD) of 1.5 × 10^−3^ ng/mL, whereas the M-ERNS-based system exhibited an LOD of 3.5 × 10^−1^ ng/mL, corresponding to an approximately 230-fold improvement in sensitivity for O-ERNS.

This substantial sensitivity enhancement is interpreted as a synergistic effect arising from both the higher single-particle SERS activity of O-ERNS and the increased probability of biomarker capture enabled by their improved antibody conjugation efficiency.

Specifically, the stronger intrinsic Raman signal of O-ERNS enhances signal readout at low analyte concentrations, while their greater outer Ag surface accessibility facilitates higher antibody loading and more efficient probe accumulation on the test line.

These results clearly demonstrate that the internal incorporation of RLCs into ERNS not only enhances SERS signal intensity but also confers structural advantages for antibody conjugation, ultimately leading to superior analytical performance in SERS-LFIA. Overall, O-ERNS represent a significantly more efficient nanoprobe design than M-ERNS for high-performance SERS-LFIA applications.

### 3.4. Dual Cancer Detection SERS-LFIA Platform for Simultaneous Detection of PSA and CA19-9 Using ERNS

**Figure 5:**
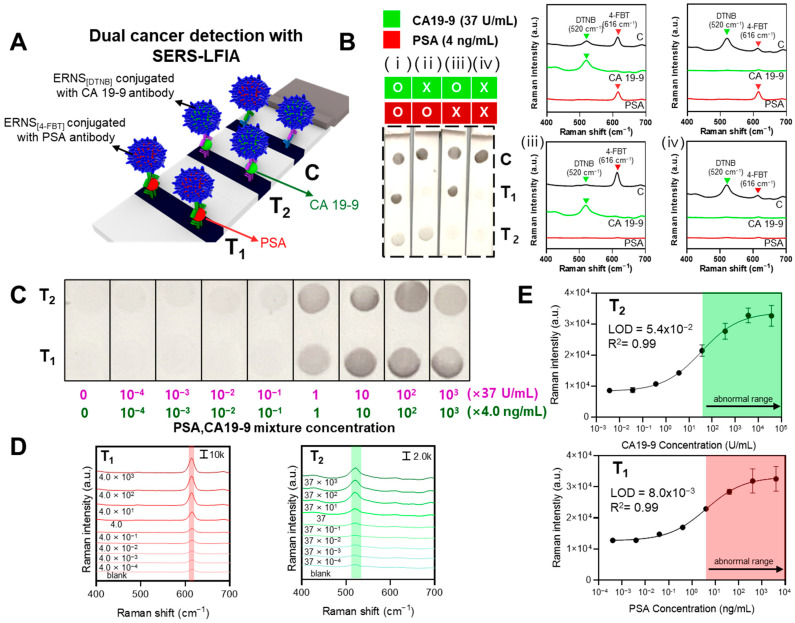
(**A**) Schematic illustration of the dual cancer detection SERS-LFIA system using ERNS labeled with different RLCs and conjugated with PSA- and CA19-9-specific antibodies. (**B**) LFIA strip images and Raman spectra obtained from samples containing PSA (4 ng/mL) and CA19-9 (37 U/mL) under four conditions: (i) PSA and CA19-9 together, (ii) PSA only, (iii) CA19-9 only, and (iv) blank sample. (**C**) Colorimetric images of test line 1 (PSA) and test line 2 (CA19-9) obtained after applying mixed samples with initial concentrations of PSA (4 × 10^3^ ng/mL) and CA19-9 (37 × 10^3^ U/mL), followed by tenfold serial dilutions. (**D**) Raman spectra collected from test line 1 and test line 2 at different concentrations of PSA and CA19-9. (**E**) Raman intensities measured at 613 cm^−1^ (4-FBT) from test line 1 (PSA) and 520 cm^−1^ (DTNB) from test line 2 (CA19-9) and the corresponding limits of detection (LOD).

Next, a dual cancer detection SERS-LFIA system capable of simultaneously detecting PSA and CA19-9 on a single strip was constructed using ERNS labeled with different RLCs, and its analytical performance was evaluated. ERNS were labeled with 4-fluorobenzenethiol (4-FBT) and DTNB, followed by conjugation with PSA- and CA19-9-specific antibodies, respectively, enabling selective detection of PSA at test line 1 and CA19-9 at test line 2 (Figure 5A).

The specificity of the system was first evaluated by performing LFIA under four different conditions: mixed samples containing both PSA (4 ng/mL) and CA19-9 (37 U/mL), samples containing only one of the two antigens, and blank samples without either antigen (Figure 5B). Visual inspection of the LFIA strips revealed colorimetric signals exclusively at the corresponding test lines when the target antigen was present, with no observable nonspecific signals. Furthermore, Raman analysis performed at test line 1, test line 2, and the control line under each condition showed that selective Raman signals were detected only at the test line corresponding to the antigen present in the sample. These results demonstrate that the system operates reliably without cross-interference between PSA and CA19-9. To further verify assay specificity in complex biological matrices, additional control experiments were conducted in the presence of non-target biomarkers.

LFIA strip images and corresponding Raman spectra were obtained under four conditions: (**i**) PSA and CA19-9 together, (**ii**) PSA only, (**iii**) CA19-9 only, and (**iv**) a blank sample, while non-target proteins were present in all cases. These results confirm that both the colorimetric and SERS responses remained exclusively specific to the target biomarkers without observable cross-interference, even under complex matrix conditions (Figure S3).

Subsequently, the simultaneous detection performance was quantitatively evaluated using mixed samples containing both PSA and CA19-9. A solution containing PSA (4 × 10^3^ ng/mL) and CA19-9 (37 × 10^3^ U/mL) was used as the highest concentration, followed by tenfold serial dilutions, and LFIA was performed to compare the resulting images at test line 1 and test line 2 (Figure 5C). As a result, clear colorimetric signals were visually observable for both PSA and CA19-9 down to concentrations approximately tenfold lower than their respective clinical cut-off values (PSA, 4 ng/mL; CA19-9, 37 U/mL) [50,51].

Raman spectra collected from the probes captured at the test lines showed concentration-dependent increases in the characteristic Raman peaks of the corresponding RLCs for PSA and CA19-9 (Figure 5D). Raman intensities measured at 613 cm^−1^ (4-FBT) and 520 cm^−1^ (DTNB) were subsequently used to evaluate analytical performance (Figure 5E). The limits of detection (LOD) were determined to be 8.0 × 10^−3^ ng/mL for PSA and 5.4 × 10^−2^ U/mL for CA19-9, corresponding to approximately 500-fold and 685-fold lower concentrations than their respective clinical cut-off values.

Overall, this dual cancer detection SERS-LFIA system enables rapid visual screening of PSA and CA19-9 at or near clinically relevant threshold levels, while Raman-based analysis allows simultaneous quantitative detection of both biomarkers down to ultralow concentrations. These results highlight the strong potential of the proposed platform as an early diagnostic system capable of high-sensitivity and high-accuracy multiplex cancer biomarker detection. In the present study, multiplex demonstration was intentionally limited to a duplex configuration due to considerations related to assay compatibility and the avoidance of cross-reactivity under a unified LFIA format. Nevertheless, because each ERNS is encoded with a spectrally distinct Raman fingerprint through RLC selection, the platform is inherently scalable to triplex or higher-order multiplex detection by incorporating additional ERNS probes.

## 4. Conclusions

This study demonstrates that a design strategy involving the internal incorporation of Raman labeling compounds (RLCs) within silica-templated ERNS can directly translate into enhanced SERS signal utilization and improved immunoassay performance. In particular, introducing RLCs into the internal structure during Ag shell growth was shown to significantly improve the efficiency with which SERS signals are exploited under plasmonic conditions. Beyond signal enhancement, this internal RLC incorporation strategy provides structural advantages for immunoassay applications by effectively preserving accessible metal surface areas for surface functionalization. As a result, a dual-mode colorimetric–SERS diagnostic concept was realized, enabling rapid visual screening through colorimetric readout together with highly sensitive quantitative analysis via Raman spectroscopy within a single platform. Furthermore, this work presents a versatile nanoprobe design framework that is not limited to a specific labeling compound or a single target biomarker, but is readily extendable to various RLCs and biomarkers. Such a structure-driven design approach offers a meaningful guideline for the development of multiplex biomarker detection systems and liquid biopsy-based diagnostic platforms, including those that may be adapted for point-of-care use in the future (Table S1).

## Data Availability

The original contributions presented in this study are included in the article/Supplementary Material. Further inquiries can be directed to the corresponding author.

## Supplementary Materials

The following supporting information can be downloaded at https://www.mdpi.com/article/10.3390/bios16020129/s1. Figure S1: Supplementary validation of RLC localization and surface accessibility of O-ERNS, Figure S2: Comparative Raman analysis of O-ERNS, M-ERNS, and a bare Ag shell to validate the relative SERS enhancement under identical experimental conditions, Figure S3: LFIA strip images and Raman spectra obtained from samples containing PSA (4 ng/mL) and CA19-9 (37 U/mL) under four conditions: (i) PSA and CA19-9 together, (ii) PSA only, (iii) CA19-9 only, and (iv) blank sample. In all cases, ICAM-1 (391 ng/mL) and APOA-1 (1 mg/mL) were consistently included as non-target interferents; Table S1: Comparative summary of recently reported visual–SERS dual-mode LFIA platforms and the present ERNS-based system.
