# Supplementary material for: Multiplexed Detection of Cancer Biomarker Using a Dual-Mode Colorimetric-SERS Lateral Flow Immunoassay Based on Elongated Rod Ag Nanoshell (ERNS) SERS Tags
Source: Biosensors (Basel). 2026 Feb 21;16(2):129. doi: 10.3390/bios16020129 (PMC12939106; doi:10.3390/bios16020129)
Supplement: Supplementary file 1 [file biosensors-16-00129-s001.zip › biosensors-4122491-supplementary.pdf]

Supporting Information

Multiplexed Detection of Cancer Biomarker Using a Dual-Mode Colorimetric-SERS Lateral Flow Immunoassay Based on Elongated Rod Ag Nanoshell (ERNS) SERS Tags

Sungwoo Park [1], Yeonghee Jeong [1], Sohyeon Jang [1], Cho-Hee Yang [1], Jun-Sik Chu [1], Homan Kang [2], Seung-min Park [3], Hyejin Chang [4] and Bong-Hyun Jun [1,*]

[1] Department of Bioscience and Biotechnology, Konkuk University, Seoul 05029, Republic of Korea; tjddn939@konkuk.ac.kr (S.P.); jyh3077@konkuk.ac.kr (Y.J.); thgus03030@konkuk.ac.kr (S.J.); vltizk0052@konkuk.ac.kr (C.-H.Y.); cjs9719@naver.com (J.-S.C.)

[2] Department of Radiology, Gordon Center for Medical Imaging, Massachusetts General Hospital and Harvard Medical School, Boston, MA 02114, USA; hkang7@mgh.harvard.edu

[3] School of Chemistry, Chemical Engineering and Biotechnology, Nanyang Technological University, Singapore 637459, Singapore; park.seungmin@ntu.edu.sg

[4] Division of Science Education, Kangwon National University, Chuncheon 24341, Republic of Korea; hjchang@kangwon.ac.kr

* Correspondence: bjun@konkuk.ac.kr; Tel.: +82-2-450-0521

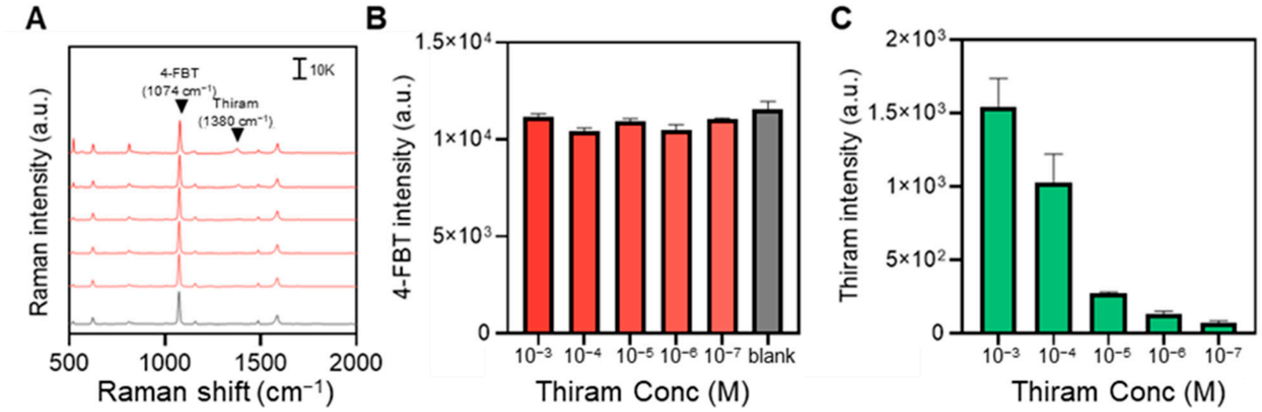

Figure S1. Supplementary validation of RLC localization and surface accessibility of O-ERNS. (**A**) Raman spectra of O-ERNS pre-labeled with 4-FBT after introduction of thiram at different concentrations. (**B**) Corresponding Raman intensity of 4-FBT on O-ERNS as a function of thiram concentration, showing negligible change compared to the blank control. (**C**) Raman intensity of thiram adsorbed on O-ERNS as a function of thiram concentration, demonstrating concentration-dependent surface binding.

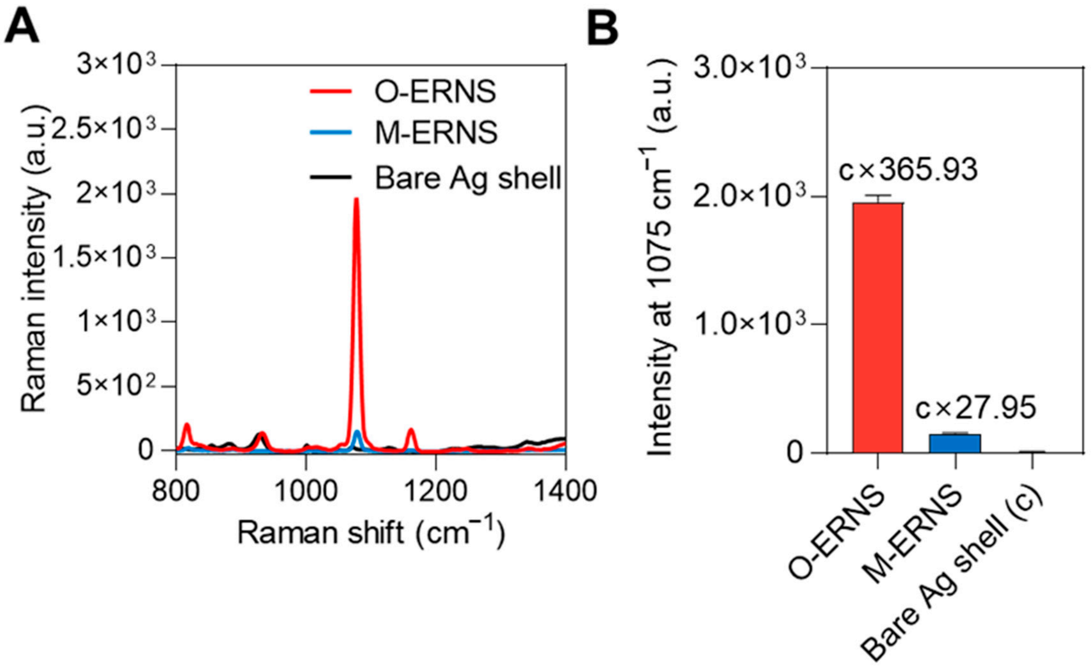

Figure S2. Comparative Raman analysis of O-ERNS, M-ERNS, and a bare Ag shell to validate the relative SERS enhancement under identical experimental conditions. (A) Raman spectra of the bare Ag shell, M-ERNS, and O-ERNS measured under identical conditions after labeling with 4-fluorobenzenethiol (4-FBT). (B) Quantitative comparison of Raman intensities at the characteristic 4-FBT peak (1075 cm^{-1}) for the bare Ag shell, M-ERNS, and O-ERNS, demonstrating a 365.93-fold enhancement for O-ERNS and a 27.95-fold enhancement for M-ERNS relative to the bare Ag shell. These results confirm an approximately 13.1-fold higher SERS signal for O-ERNS compared with M-ERNS

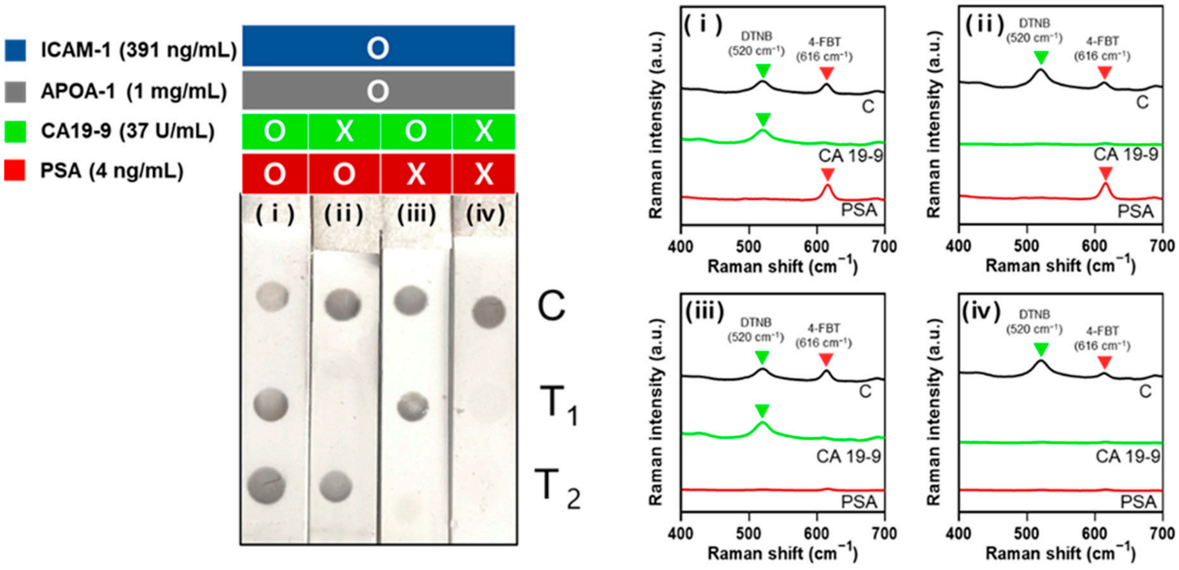

Figure S3. LFIA strip images and Raman spectra obtained from samples containing PSA (4 ng/mL) and CA19-9 (37 U/mL) under four conditions: (i) PSA and CA19-9 together, (ii) PSA only, (iii) CA19-9 only, and (iv) blank sample. In all cases, ICAM-1 (391 ng/mL) and APOA-1 (1 mg/mL) were consistently included as non-target interferents. The results demonstrate clear target-specific colorimetric and SERS responses without cross-interference, even in the presence of excess non-target biomarkers.

Table S1. Comparative summary of recently reported visual–SERS dual-mode LFIA platforms and the present ERNS-based system.

	A	B	C	D	E	F	G	H	I	J	Current work
**Raman analysis instrumentation**	Conventional Raman	Conventional Raman	Conventional Raman	Raman mapping	Raman mapping	Raman mapping	Raman mapping	**Portable Raman**	**Portable Raman**	**Portable Raman**	**Portable Raman**
**NP synthesis and Raman label incorporation process**	Multi-step	-	Multi-step	Multi-step	Multi-step	Multi-step	Multi-step	Multi-step	Multi-step	Multi-step	**One-step**
**Antigen-specific Raman labeling**	No	No	No	**Yes**	**Yes**	**Yes**	No	No	No	**Yes**	**Yes**
**Antigen discrimination at each test line**	No	No	No	No	No	**Yes**	No	No	No	No	**Yes**
**Resistance to non-specific binding interference in Raman quantification**	Limited	Limited	Limited	Limited	Limited	**High**	Limited	Limited	Limited	Limited	**High**
**Number of test lines**	**2**	**2**	**3**	**2**	**2**	**2**	**3**	1	**2**	**3**	**2**
**Target disease**	Foodborne pathogens	Veterinary antibiotics	Environmental contaminants	Hospital-acquired pathogens	Veterinary drug residues	Cardiovascular biomarkers	Myocardial injury markers	Protein toxin–based diseases	Viral infection biomarkers	Agricultural mycotoxins	**Multi-cancer biomarker**
**References**	Liu H.-B. et al., J. Agric. Food Chem., 65 (2017) 10290–10299	Shi Q. et al., Microchim. Acta, 185 (2018) 1–8	Wang J. et al., Sci. Total Environ., 912 (2024) 169440	Wang C. et al., Biosens. Bioelectron., 214 (2022) 114525	Tu J. et al., J. Hazard. Mater., 448 (2023) 130912	Cao X. et al., Nanotechnology, 32 (2021) 445101	Zhang D. et al., Biosens. Bioelectron., 106 (2018) 204–211	Jia X. et al., Nanomedicine, 41 (2022) 102522	Li Y. et al., Nanomaterials, 11 (2021) 6	Zhang W. et al., J. Hazard. Mater., 393 (2020) 122348	
